# IgE Reactivity of Blue Swimmer Crab (*Portunus pelagicus*) Tropomyosin, Por p 1, and Other Allergens; Cross-Reactivity with Black Tiger Prawn and Effects of Heating

**DOI:** 10.1371/journal.pone.0067487

**Published:** 2013-06-19

**Authors:** Jodie B. Abramovitch, Sandip Kamath, Nirupama Varese, Celia Zubrinich, Andreas L. Lopata, Robyn E. O'Hehir, Jennifer M. Rolland

**Affiliations:** 1 Department of Immunology, Monash University, Melbourne, Victoria, Australia; 2 Department of Allergy, Immunology and Respiratory Medicine, The Alfred Hospital and Monash University, Melbourne, Victoria, Australia; 3 School of Pharmacy and Molecular Science, James Cook University, Townsville, Queensland, Australia; Cordelier Research Center, INSERMU872-Team16, France

## Abstract

Shellfish allergy is a major cause of food-induced anaphylaxis, but the allergens are not well characterized. This study examined the effects of heating on blue swimmer crab (*Portunus pelagicus*) allergens in comparison with those of black tiger prawn (*Penaeus monodon*) by testing reactivity with shellfish-allergic subjects' serum IgE. Cooked extracts of both species showed markedly increased IgE reactivity by ELISA and immunoblotting, and clinical relevance of IgE reactivity was confirmed by basophil activation tests. Inhibition IgE ELISA and immunoblotting demonstrated cross-reactivity between the crab and prawn extracts, predominantly due to tropomyosin, but crab-specific IgE-reactivity was also observed. The major blue swimmer crab allergen tropomyosin, Por p 1, was cloned and sequenced, showing strong homology with tropomyosin of other crustacean species but also sequence variation within known and predicted linear IgE epitopes. These findings will advance more reliable diagnosis and management of potentially severe food allergy due to crustaceans.

## Introduction

Shellfish play an important role in human nutrition and health, but can provoke serious IgE-mediated adverse reactions in susceptible individuals. Shellfish are a major cause of food-induced anaphylaxis [1]–[3]. Currently, there is no specific therapy for shellfish allergy, with only emergency treatment following accidental exposure [4]. Unlike most food allergies, allergy to shellfish is typically life-long and predominantly affects the adult population [5]–[6]. A major difficulty in managing shellfish allergy is the lack of reliable diagnostic assays due to limited knowledge of clinically relevant shellfish allergens.

The shellfish group includes crustaceans (phylum arthropoda including prawns, lobsters and crabs) and molluscs (phylum mollusca including oysters, mussels and squid). In the few studies characterizing shellfish allergens to date, including our own, one of the most frequently recognized (major) allergens of species in both shellfish phyla is the abundant muscle protein tropomyosin (TM) [7]–[11]. Other identified allergens are also derived from muscle tissue: myosin light-chain, arginine kinase, sarcoplasmic Ca-binding protein and troponin C [9], [11]–[14]. However, only a few species have been studied to date, mostly shrimp and prawn, with few reports on crab allergens [11], [15]. Prior to our current report, only one crab allergen, TM from the crucifix crab (*Charybdis feriatus*), was published in the International Union of Immunological Societies (IUIS) allergen database (http://www.allergen.org/index.php). Furthermore, there is little information on shellfish from the southern hemisphere or Asia-Pacific region. Patients frequently report clinical reactions to more than one shellfish species, but whether this is a result of multiple sensitivities or from IgE cross-reactivity between allergens of different shellfish species is unknown [5], [16]. This information is vital for optimal management of shellfish allergy.

Adding complexity, there are reports of altered stability and allergenicity of food proteins after processing [17]–[19]. Most members of the TM allergen family are highly heat-stable [20]–[21]. However, there is a paucity of information on the effects of heating on allergens within whole shellfish extracts [22], with most studies testing heated purified allergens. Heating can enhance allergenicity through several mechanisms including protein denaturation and exposure of new epitopes, aggregation and chemical modification such as the Maillard reaction [23].

We report here the characterization of allergenicity of a commonly eaten crustacean species, the blue swimmer crab (*Portunus pelagicus*), and in particular the identification of the major allergen Por p 1. Evidence of cross-species IgE reactivity with another commonly consumed species, the black tiger prawn (*Penaeus monodon*), was sought and the effect of heating on allergens of both species and their cross-reactivity was assessed. Clinically relevant IgE reactivity to the shellfish extracts was assessed by a whole blood basophil activation assay.

## Materials and Methods

### Ethics Statement

Informed written consent was obtained from all subjects, with ethics approvals from the Alfred Hospital Research Ethics Committee (Project number 192/07) and the Monash University Human Ethics Committee (MUHREC CF08/0225).

### Study Population and Sera

Serum samples were obtained from twenty-four shellfish-allergic subjects (mean age 32±10.5 years; 13/24 female), seven non-atopic controls (mean age 40.3±12.3 years; 4/7 female) and one atopic non-shellfish-allergic subject (age 28 years, female). Allergic subjects were identified from the Alfred Hospital Allergy clinic seafood allergy database on the basis of clinical history of allergy to shellfish and positive shrimp-specific IgE (ImmunoCAP [Phadia Pty Ltd, Uppsala, Sweden] >0.35 kU_A_/L) (Table 1). Of these subjects, 18/24 (75%) were also positive for crab-specific IgE. Eight control subjects were selected on the basis of no clinical history of shellfish allergy; seven were non-atopic, i.e. had a negative skin prick test response to a panel of common aeroallergens and one was atopic (Bahia grass pollen-sensitized).

**Table 1:** Clinical features of subjects with allergy to shellfish.

Subject	Age (yrs)	Sex	Total IgE (IU/mL)	Crab specific IgE (kU_A_/L)	Shrimp specific IgE (kU_A_/L)	Clinical presentation to shellfish	
						Symptoms	Known shellfish species
1	19	F	242	1.17	1.32	As, R, U	Prawn
2	45	M	136	2.77	4.54	O	Prawn
3	25	M	158	0.57	0.85	R, U, An	Prawn
4	28	F	238	6.05	5.93	A, An	Prawn
5	23	M	283	18.1	19.7	As, R, O, U	Prawn
6	22	F	3401	3.36	6.65	As, R, U, An	Prawn
7	47	M	822	2.72	5.37	A, U	Prawn and all crustaceans
8	24	F	1946	9.5	2.42	R, A, O	Prawn, crab
9	35	F	3887	2.94	3.33	O, U	Shrimp
10	39	M	192	0.06	1.22	R, O	Raw prawn
11	44	M	976	8.21	9.03	R, O	Squid
12	23	M	658	4.56	5.14	R, U, An	Prawn
13	26	M	127	0.15	1.65	O	Prawn
14	55	F	566	0.13	1.36	As, R, O	Raw prawn, crab
15	37	M	194	0.2	1.41	R, O	Prawn, lobster, crayfish
16	47	F	92	0.01	1.41	R, O	Prawn, lobster
17	32	F	28	8.42	9.82	O, U	Shellfish
18	30	F	748	0.63	2.84	R, U	Crustaceans
19	32	M	183	3.09	6.84	An, O	Crustaceans
20	22	F	164	0.01	1.43	As, R	Prawn
21	17	F	1550	58.3	60.8	U, An	Prawn
22	22	M	81	2.37	2.57	As, R, U, An, A	Crustaceans
23	44	F	167	22.9	32.4	As, R, U	Prawn
24	31	F	130	6.97	8.98	O	Crab

F: female, M: male. As: asthma, R: rhinitis, A: anaphylaxis, U: urticaria, An: angioedema, O: oral/facial symptoms.

### Preparation of Shellfish Extracts

Fresh blue swimmer crab (*Portunus pelagicus*) and black tiger prawn (*Penaeus monodon*) were purchased at Prahran market (Melbourne, Australia). For raw crab (RC) and raw prawn (RP) extracts, the outer shell was removed and muscle collected. Finely cut muscle was blended with PBS pH 7.2 and left overnight at 4°C with constant mixing. The crude extract was centrifuged at 13,000 rpm at 4°C for 20 minutes. Supernatant was collected and filter sterilized before storage at −80°C in aliquots. For cooked crab (CC) and cooked prawn (CP) extracts, the outer shell was kept during the heating process (20 minutes immersed in boiling PBS) before removal and extract preparation as above. The protein concentration of each extract was determined using the Bradford assay kit (Bio-Rad Laboratories, Hercules, CA) using bovine gamma globulin as a standard.

### Sodium Dodecyl Sulfate-Polyacrylamide Gel Electrophoresis (SDS-PAGE)

Proteins of shellfish extracts, 15 µg/lane, were separated by electrophoresis under reducing conditions using 4–12% Bis-Tris gels (NuPage, Carlsbad, CA). Pre-stained standards (1× See Blue Plus2, Invitrogen, Carlsbad, CA) were used as molecular weight (MW) markers. Proteins were resolved at 200 V for 35 minutes using an Xcell II mini-cell apparatus (Invitrogen) and the gel was stained with Coomassie brilliant blue.

### IgE ELISA and Inhibition IgE ELISA

Wells of a 96-well EIA/RIA plate (Costar, St. Louis, MO) were coated with 100 µL extract (RC, CC, RP or CP; 1 µg/mL in PBS, or PBS alone for ‘no-antigen’ control wells), and incubated overnight at 4°C. All of the following incubations were performed for 1 hour unless otherwise stated and the plate was washed 4 times in 0.05% Tween 20/PBS (PBS-T) between steps. Blocking was performed using 5% skim milk powder diluted in PBS-T. Serum, 100 µL diluted 1∶10 in 1% skim milk powder/PBS-T, was added to wells and then incubated for 3 hours at room temperature with shaking (45 rpm). Rabbit anti-human IgE antibody (1∶4000; Dako, Glostrup, Denmark) and goat anti-rabbit IgG-HRP (1∶1000; Promega, Madison, WI) were added sequentially to wells and plates incubated at room temperature for 1 hour with gentle shaking. Plates were then washed 5 times in PBS-T, followed by 3 washes in PBS. IgE binding was detected using TMB (3,3′,5,5′-Tetramethylbenzidine) substrate (Invitrogen). After 5 minutes, the reaction was terminated using 1 M HCl and the absorbance (O.D.) at 450 nm measured by spectrophotometry (Thermo Fisher Scientific, Melbourne, Australia). Sera from seven non-atopic subjects were screened to determine the extent of non-specific binding. ‘No-antigen’, background values were subtracted from test sera data. Two standard deviations above the mean O.D._450 nm_ value for the non-atopic subject sera were used to determine the cut-off for positive IgE binding in shellfish-allergic subjects.

For inhibition ELISA experiments, individual subject sera were first titrated (1∶10–1∶1280) for IgE reactivity with CC or CP to determine the concentration at which the O.D._450 nm_ was ∼1 and within the linear phase of the titration curve. Using this dilution, serum samples were incubated with an equal volume of shellfish extract (RC, CC, RP or CP at 0.16, 0.8, 4, 20 and 100 µg/mL), purified recombinant TM from black tiger prawn (rPen m 1.0101; Kamath et al., unpublished) or purified recombinant TM from blue swimmer crab (rPor p 1) (at 0.048, 0.24, 1.2, 6, and 30 µg/mL) for 1 hour at room temperature and then tested for reactivity with either CC or CP extracts. Percent inhibition was calculated using the following equation: percent inhibition  = 100−[(O.D._450 nm_ of serum with inhibitor/O.D._450 nm_ of serum without inhibitor) X100]. For comparison of inhibition by different extracts, the extract (inhibitor) concentration that caused 50% inhibition was calculated from dose-response curves. To assess non-specific inhibition by extracts, serum from a non-shellfish-allergic, atopic (Bahia grass pollen-sensitized) subject was incubated with the above inhibitors, and then tested for IgE reactivity with Bahia grass pollen extract in comparison with untreated serum.

### IgE Immunoblot and Inhibition IgE Immunoblot

Proteins of each of the four extracts (RC, CC, RP, CP) were separated by SDS-PAGE as above with 3 µg/well loaded into a 10 or 15-well 4–12% Bis-Tris gel (NuPage), or 60 µg protein loaded into a 6 cm 2D well 4–12% Bis-Tris gel (NuPage). Proteins were transferred to a nitrocellulose membrane (0.45 µm; Thermo Scientific, Rockford IL) using an Xcell II blotting apparatus (Invitrogen) at a constant voltage of 30 V for 1 hour. Successful transfer of protein was evaluated by Coomassie brilliant blue staining of the gel as above. The membrane was blocked using 5% skim milk powder/PBS-T for 1 hour at room temperature with gentle rocking. Subject serum (1∶40 in 5% SMP/PBS-T) was then applied to the membrane using a Miniblotter apparatus (Immunetics, Boston, MA). To detect IgE binding, the immunoblot was incubated sequentially with rabbit anti-human IgE antibody (Dako; 1∶15000) and goat anti-rabbit IgG-HRP conjugated antibody (Promega; 1∶15000) each for 1 hour at room temperature with gentle horizontal shaking. Following incubation with chemiluminescent peroxidase substrate (Sigma-Aldrich, St. Louis, MO), proteins were visualized using enhanced chemiluminescence technique [24]. TM bands were identified in parallel immunoblots using a rat anti-TM monoclonal antibody (mAb; 1∶6000; Abcam, Cambridge, UK) followed by rabbit anti-mouse IgG-peroxidase antibody (1∶80000; Sigma-Aldrich) and development as above.

For inhibition IgE immunoblot experiments, serum samples (final concentration 1∶40) were first incubated with whole crude extracts (RC, CC, RP or CP at 4, 20 and 100 µg/mL) or rPen m 1 (1.2 µg/mL) for 1 hour at room temperature and then tested for reactivity with the CC or CP extracts by immunoblotting as described above. In a separate experiment to directly compare rPor p 1 with rPen m 1, serum samples were incubated with rPor p 1 or rPen m 1 at 0.048, 0.24 and 1.2 µg/mL. A ‘no inhibitor’ control was also included. Complete protease inhibitor (Roche, Basel, Switzerland) was added to the diluent (one tablet in 50 mL of diluent) to prevent non-specific proteolytic degradation of IgE antibodies. Non-specific inhibition was assessed as described for the inhibition IgE ELISA above.

### Sequence Analysis of Blue Swimmer Crab Tropomyosin

A band corresponding to the predominant IgE-reactive 39 kDa protein was excised from the SDS gel for mass spectrometric analysis. The band was de-stained, reduced, alkylated and digested with trypsin as reported previously [9]. Digested protein was injected into a DIONEX Ultimate 3000 liquid chromatography system (Germering, Germany) coupled with a QSTAR XL hybrid quadrupole-quadrupole/time-of-flight tandem mass spectrometer (QqToF-MS/MS; Applied Biosystems/MDS Sciex, Foster City, USA). The resultant tandem spectra were searched using the Matrix Science (Mascot) search engine (precursor and product ion mass tolerance set at 0.1 Da). For cloning and full sequencing of the crab TM, total RNA was extracted from crab pincer muscles using Trizol reagent (Invitrogen) following the manufacturer's instructions. Single stranded cDNA was reverse transcribed from the RNA using RT-PCR with a cDNA synthesis kit (Bioline, Sydney, Australia). Oligo(dT) primers were used for the reverse transcription. Due to the high amino acid sequence identity of the N- and C-terminal regions of TM among crustacean species, the primers were designed based on the amino acid sequence of Rock lobster TM, previously determined by our group (Rock lobster, *Jasus lalandii*, Genbank accession number JX860677.1). The primer pair used was BSC-TM (F) 5′-GCCGGATCC-ATGGACGCAATCAAGAAGAAGATGCAG-3′ and BSC-TM (R) 5′-GCGGAATTC-TTAGTAGCCAGACAGTTCG-3′. The PCR included one cycle of denaturation at 94°C for 2 minutes, 35 cycles at 94°C for 30 seconds, annealing at 55°C for 45 seconds and elongation at 72°C for 1 minute, and a final elongation step at 72°C for 7 minutes. The amplified full length open reading frame was cloned into the sequencing vector, pCR 2.1 (Invitrogen) using the BamH1 and EcoR1 restriction sites for cloning, and the open reading frame for TM sequenced (Macrogen Inc, Seoul, South Korea) to obtain the construct pCR2.1_TM.

### Expression and Purification of Recombinant Blue Swimmer Crab Tropomyosin

The open reading frame of TM was cross-cloned from the construct, pCR2.1_TM to the expression vector, pProEXHT-B using restriction sites for BamH1 and EcoR1 and ligation was performed using T4 DNA Ligase (Invitrogen, CA, USA). This expression plasmid construct was transformed into chemical-competent NM522 *E.coli* cells using heat-shock for 30 seconds, incubation in SOC medium at 37°C for 1 hour and grown overnight on Luria Bertani (LB) agar plates with 100 µg/mL ampicillin (Amresco, USA) at 37°C. Positive colonies were tested using PCR as described above and selected for protein expression.

For recombinant protein expression, 5 mL of fresh overnight culture from a single colony was used to initiate growth in 1 L LB broth containing 100 µg/mL ampicillin. Recombinant protein expression was induced using 0.6 mM isopropylthio-β-galactoside (Amresco, USA). After expression, the culture was centrifuged at 3500 g for 10 minutes to obtain the bacterial pellet and subsequently resuspended in extraction buffer (25 mM Tris-HCl, 300 mM NaCl, 1 mM imidazole, pH 8). Recombinant blue swimmer crab TM containing the 6xHis tag was extracted from the *E.coli* cells using a French-Pressure Cell, purified using nickel charged metal-chelate affinity chromatography (GE Healthcare, USA) following the manufacturer's instructions and stored at −80°C until further use. The protein concentration of the purified protein was determined by absorbance at 280 nm using a nanodrop spectrophotometer (ND-1000, NanoDrop Technologies Inc., Wilmington, Delaware, USA).

### Whole Blood Basophil Activation Test

Shellfish extracts were tested for basophil activation using our established methodology [25]. Briefly, heparinised peripheral blood samples (100 µL) from five shellfish-allergic subjects, a non-shellfish allergic atopic control and one non-atopic control were incubated with shellfish extracts (0.01–10 µg/ml) or rPen m 1 (0.001–1 µg/mL) for 20 minutes at 37°C and then basophil activation was assessed by flow cytometry by determining the percentage of viable (7-AAD negative), high IgE-positive cells expressing surface CD63. Anti-IgE antibody (cross-linking) and the bacterial peptide f-Met-Leu-Phe (fMLP) were used as positive controls (for IgE-dependent and -independent activation respectively), and stimulation buffer alone was used as a negative control.

### Statistical Analysis

The Wilcoxon matched-pairs signed rank test was used to compare overall serum IgE reactivity between shellfish extracts, and Spearman's correlation test was used to assess correlation between individual specific IgE levels against different extracts or using different assays. Analyses were performed using GraphPad Prism version 5.04 for Windows (GraphPad, San Diego, CA).

## Results

### SDS-PAGE Analysis of Shellfish Extracts

**Figure 1:**
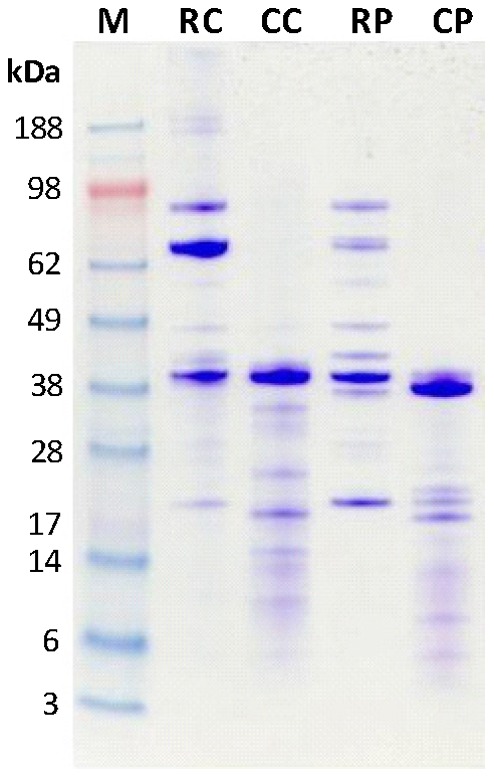
SDS-PAGE analysis of shellfish extracts. 4–12% SDS-PAGE of whole shellfish extracts stained with Coomassie brilliant blue. M, MW markers; RC, raw blue swimmer crab; CC, cooked blue swimmer crab; RP, raw black tiger prawn; CP, cooked black tiger prawn.

Analysis of raw and cooked shellfish extracts by SDS-PAGE and Coomassie brilliant blue staining (Figure 1) revealed an array of proteins ranging from ≈6 to 188 kDa. A prominent protein band at 37–39 kDa was seen in all extracts, consistent with TM (34–39 kDa). Other bands were observed at positions consistent with the known shellfish allergens arginine kinase (≈42 kDa), myosin light chain, sarcoplasmic calcium binding protein and troponin C (≈21 kDa), but several other bands were also apparent at positions that do not correspond to known shellfish allergens. Some differences could be seen between the RC and RP extracts, most notably the band at 69 kDa seen strongly in the RC but only weakly in the RP. In addition, there was only one major protein band in the TM region in RC, whilst there were two bands in RP. More pronounced differences were seen when raw and cooked extracts of both species were compared. For both CC and CP extracts, the higher MW proteins seen in the raw extracts were not present, most likely due to protein degradation during the cooking process. This is supported by the appearance of lower (<35 kDa) MW proteins only present in the cooked extracts. The actual sizes of these lower proteins differed between the crab and prawn extracts. The MW of the prominent TM region band for the prawn extract decreased from 39 kDa to 37 kDa on cooking, but did not change for the CC extract, remaining at 39 kDa.

### ELISA for Serum IgE Reactivity to Shellfish Extracts

**Figure 2:**
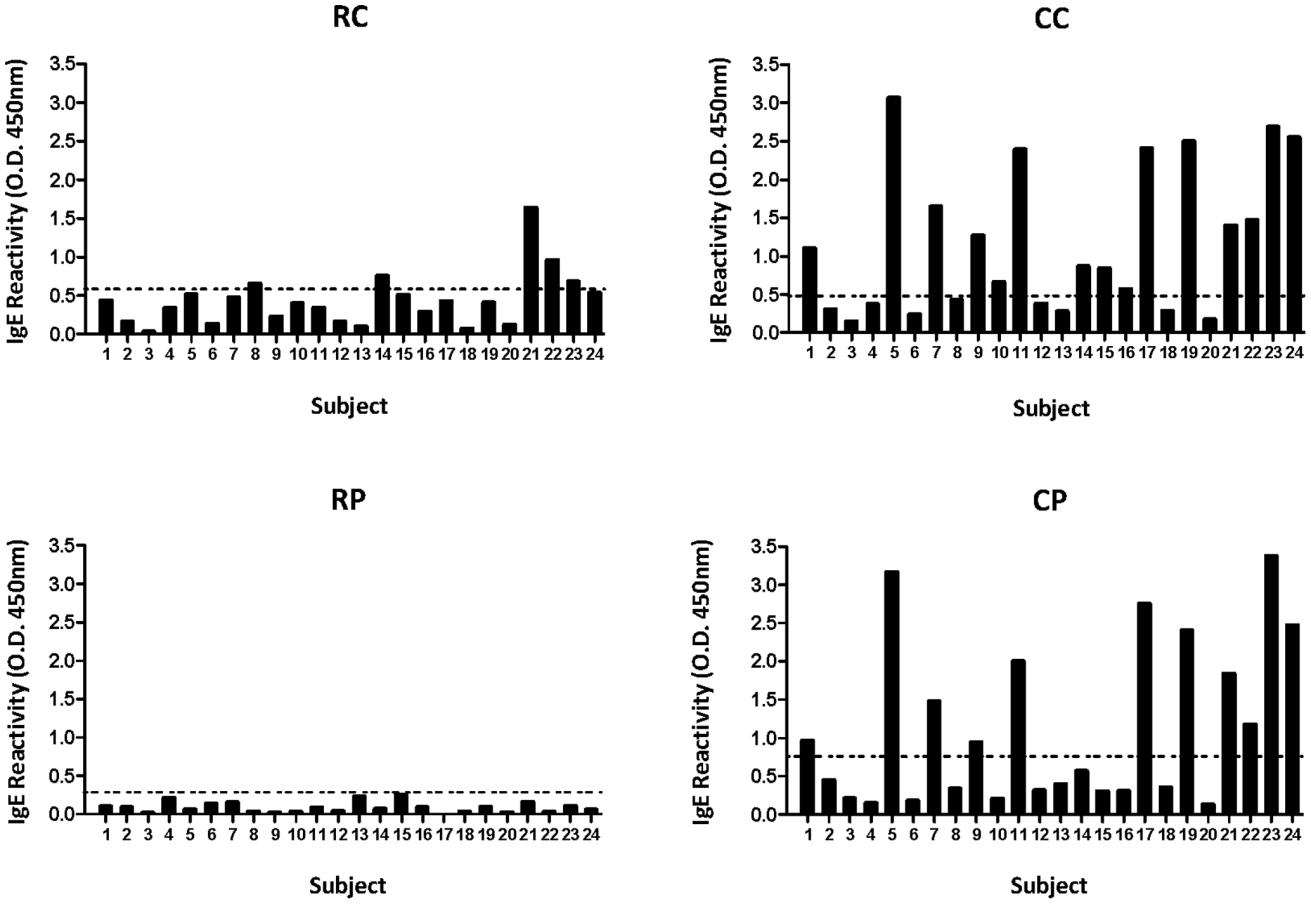
ELISA for serum IgE reactivity to shellfish extracts. ELISA for serum IgE reactivity to raw blue swimmer crab (RC), cooked blue swimmer crab (CC), raw black tiger prawn (RP) and cooked black tiger prawn (CP) for 24 shellfish-allergic subjects. The cut-off of two standard deviations above mean reactivity of 7 non-atopic subjects to each of the extracts is indicated by the dotted line (0.56 for RC, 0.49 for CC, 0.29 for RP and 0.75 for CP).

Quantitation of serum IgE binding to the shellfish extracts by ELISA (Figure 2) showed that the cooked extracts have markedly higher IgE reactivity than the corresponding raw extracts. Median O.D. values for CC and RC were 0.86 and 0.41 respectively (CC vs RC p<0.001) and for CP and RP were 0.51 and 0.08 respectively (CP vs RP p<0.001). The RC extract was significantly more IgE reactive than RP (p<0.001), but there was no overall difference between the two cooked extracts. Of the 24 shellfish-allergic subjects, 5 (21%) had positive IgE reactivity to RC, 15 (63%) to CC (including 4 of the 5 RC positives), none to RP, and 11 (46%) to CP. A similar pattern of reactivity was observed between the CC and CP extracts. All subjects who were positive to CP were also positive to CC, and of those positive to CC but not to CP, reactivity was only weak (10, 14, 15 and 16). These same four subjects had a negative crab ImmunoCAP. Overall there was a significant correlation between IgE levels by ELISA for the CC and CP and the relevant ImmunoCAP values (p<0.01), but not for the raw extracts. However, several subjects showed a lack of concordance of positive or negative result for ELISA with cooked extracts and ImmunoCAP.

### IgE Immunoblotting of Shellfish Extracts

**Figure 3:**
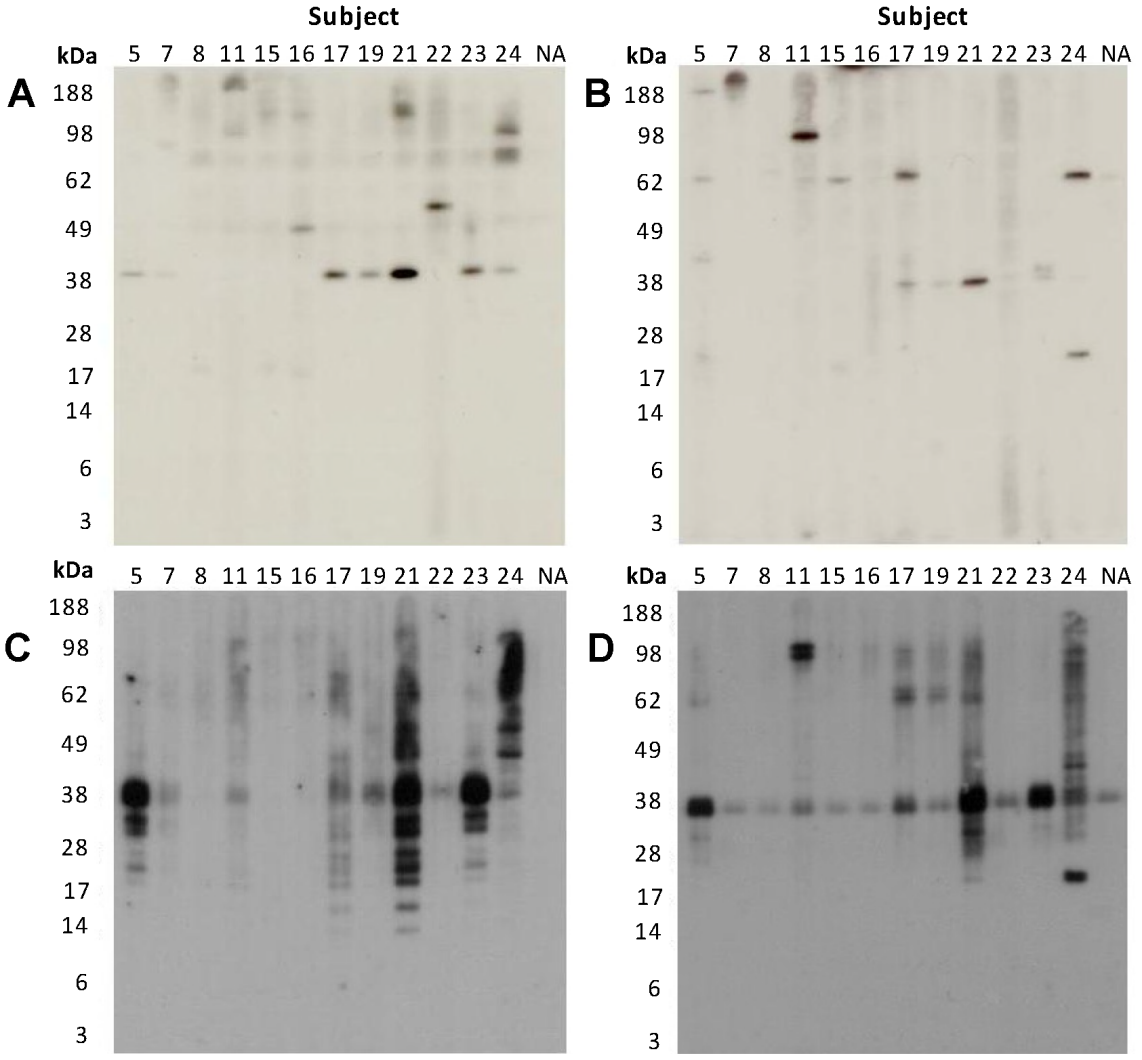
Immunoblotting for serum IgE reactivity against shellfish extracts. Sera from 12 shellfish-allergic subjects (5–24) and one non-atopic (NA) subject were tested for IgE reactivity to shellfish extract proteins separated by 4–12% SDS-PAGE. **A.** RC, **B.** RP, **C.** CC, **D.** CP.

Sera from 12 subjects with IgE positivity to RC and/or CC by ELISA, and where sufficient serum was available, were used for immunoblotting to visualize IgE-reactive proteins in the shellfish extracts (Figure 3). Immunoblotting showed markedly increased IgE binding to cooked compared with raw extracts, in terms of number of proteins recognized and intensity of binding. In particular there was increased IgE binding to proteins within the TM region (37–39 kDa); 9 (75%) subjects showed IgE binding within this region for CC (7 in RC) and 7 (58%) subjects for CP (3 in RP). The identity of the protein(s) within this region was confirmed as TM using an anti-TM mAb (data not shown). An increase in IgE-reactive lower MW proteins (<39 kDa) was observed in the CC extract and to a lesser extent in the CP extract. A protein of ≈62 kDa was recognized by 5/12 (42%) subjects in the RP extract but showed little or no reactivity in the RC or cooked extracts. For both the raw and cooked extracts, IgE-reactive proteins were observed at 40 kDa and 20–28 kDa that could correspond to other documented allergens i.e. arginine kinase, and sarcoplasmic calcium binding protein, myosin light chain and troponin C, respectively.

### Sequence Analysis of Blue Swimmer Crab Tropomyosin

**Figure 4:**
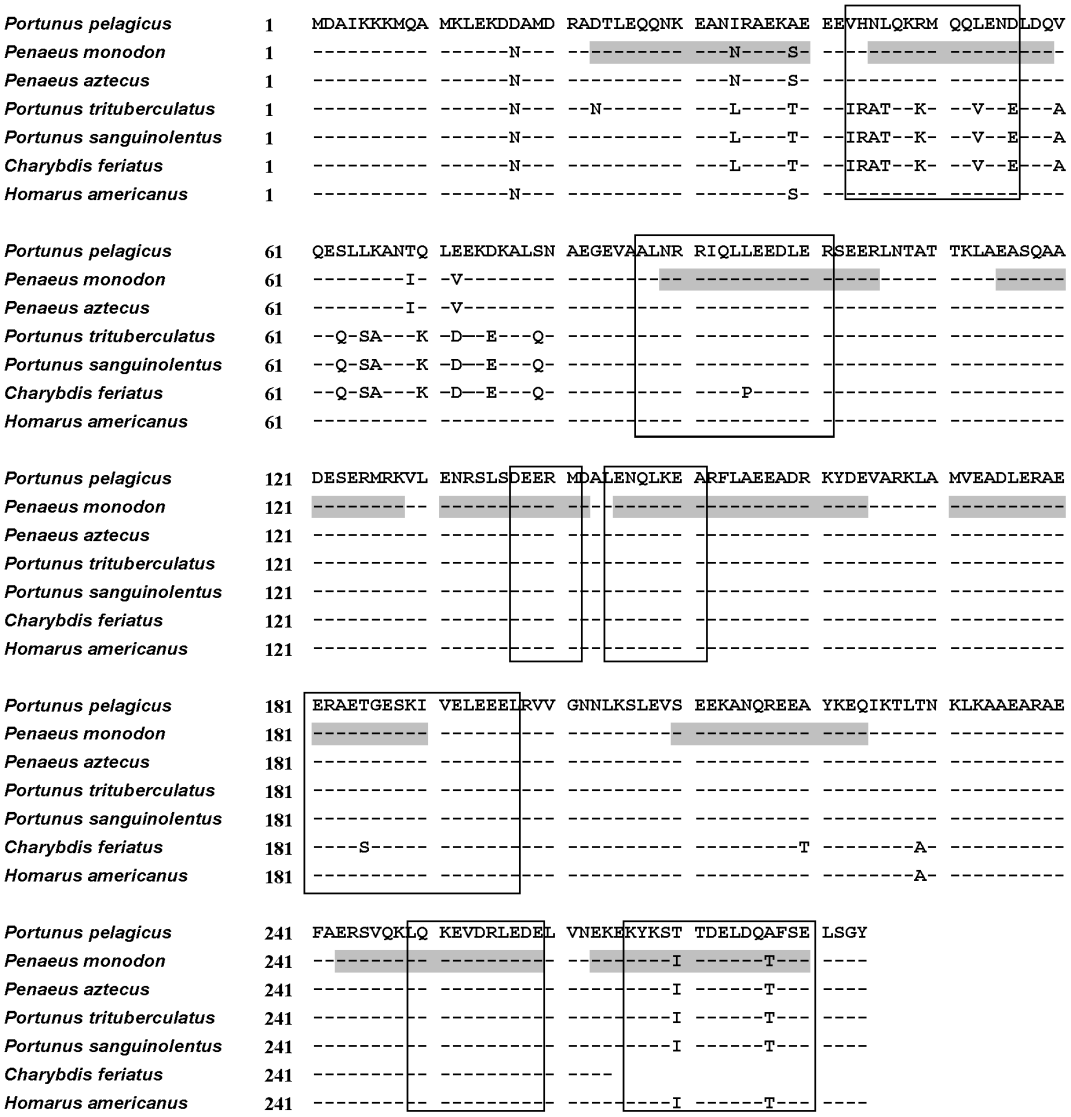
Tropomyosin sequence alignment. Alignment of TM sequences with *Portunus pelagicus* (blue swimmer crab) TM, Por p 1 (Genbank accession number JX874982.1), as reference using NCBI Protein BLAST. Species include *Penaeus monodon* (NCBI protein database accession number: A1KYZ2.1), *Portunus trituberculatus* (ABL89183.1), *Portunus sanguinolentus* (ABS12234.1), *Charybdis feriatus* (Q9N2R3.1) and *Homarus americanus* (AAC48288.1). Sequences that correlate with known linear epitopes of *Penaeus aztecus* TM (Pen a 1) [26]–[27] are boxed. Predicted linear IgE epitopes based on studies with *Penaeus monodon* TM (Pen m 1) [28] are shaded grey.

Following analysis of allergenic proteins using IgE immunoblot, the highly IgE-reactive 39 kDa protein of the blue swimmer crab was identified as TM by peptide mass fingerprinting analysis (Table 2). The blue swimmer crab TM was subsequently cloned and the complete sequence derived from cDNA and published in Genbank under accession number JX874982.1 (Figure 4). Based on the allergen sequence and patient serum IgE reactivity (see below), this allergen has been designated Por p 1.0101 by the IUIS allergen nomenclature subcommittee (http://www.allergen.org/index.php). The most similar TMs were from the American lobster (*Homarus americanus*) and the black tiger prawn (*Penaeus monodon*) which both showed 98% sequence identity. Sequence identity with other *Portunus* species TM, and the only crab TM, Cha f 1, listed within the IUIS allergen database was 92%. There were no amino acid differences between relevant regions in the blue swimmer crab TM and published linear IgE epitopes described for *Penaeus aztecus* (Pen a 1) [26]–[27] and *Penaeus monodon* (Pen m 1) [28] TMs, except for the last epitope (amino acid 266 to 280) where two amino acid substitutions were identified. Another region (amino acid 43 to 56) of known and predicted IgE epitope specificity for other crustacean species showed several amino acid differences for the blue swimmer crab (Figure 4).

**Table 2:** List of generated peptides of blue swimmer crab tropomyosin using trypsin and mass spectroscopy analysis.

Residue numbers *	Peptide sequence
77–90	ALQNAEGEVAALNR
92–101	IQLLEEDLER
141–149	MDALENQLK
168–178	KLAMVEADLER
190–198	IVELEEELR
252–264	EVDRLEDELVNEK

* Residue numbers corresponding to the full sequence of blue swimmer crab tropomyosin, Por p 1 (Genbank accession number JX874982).

### IgE Reactivity of Recombinant Blue Swimmer Crab Tropomyosin, rPor p 1

**Figure 5:**
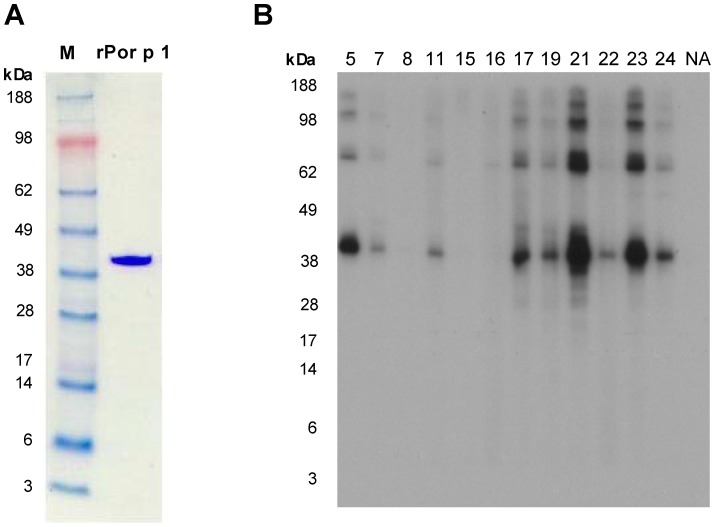
Characterization of recombinant blue swimmer crab tropomyosin, rPor p 1. **A.** 4–12% SDS-PAGE of recombinant blue swimmer crab TM, rPor p 1 stained with Coomassie brilliant blue. M, MW markers. **B.** Sera from 12 shellfish-allergic subjects (5–24) and one non-atopic (NA) subject were tested for IgE reactivity to rPor p 1 using direct IgE immunoblotting.

Recombinant blue swimmer crab TM, rPor p 1, was successfully expressed and purified with approximately 95% purity. Coomassie brilliant blue staining of SDS-PAGE of rPor p 1 under denaturing conditions showed a single band with a MW of approximately 41 kDa (Figure 5A). The higher MW of the rPor p 1 band compared to that for the natural blue swimmer crab TM in whole extract is due to the attached linker chain and 6-histidine tag. When an immunoblot was probed with sera from shellfish-allergic patients, rPor p 1 was shown to be highly IgE reactive (Figure 5B). There was strong IgE binding to the 41 kDa band for 75% of the patient sera tested, but no reactivity by a control non-atopic donor serum. Sera that showed IgE reactivity with rPor p 1 were those that reacted with the prominent TM band in the IgE immunoblots of the whole blue swimmer crab extract (Figure 3). Additional IgE-reactive higher MW bands observed in the rPor p 1 preparation are most likely multimers of rPor p 1 since their reactivity paralleled that for the major 41 kDa band.

### Basophil Activation Test

**Figure 6:**
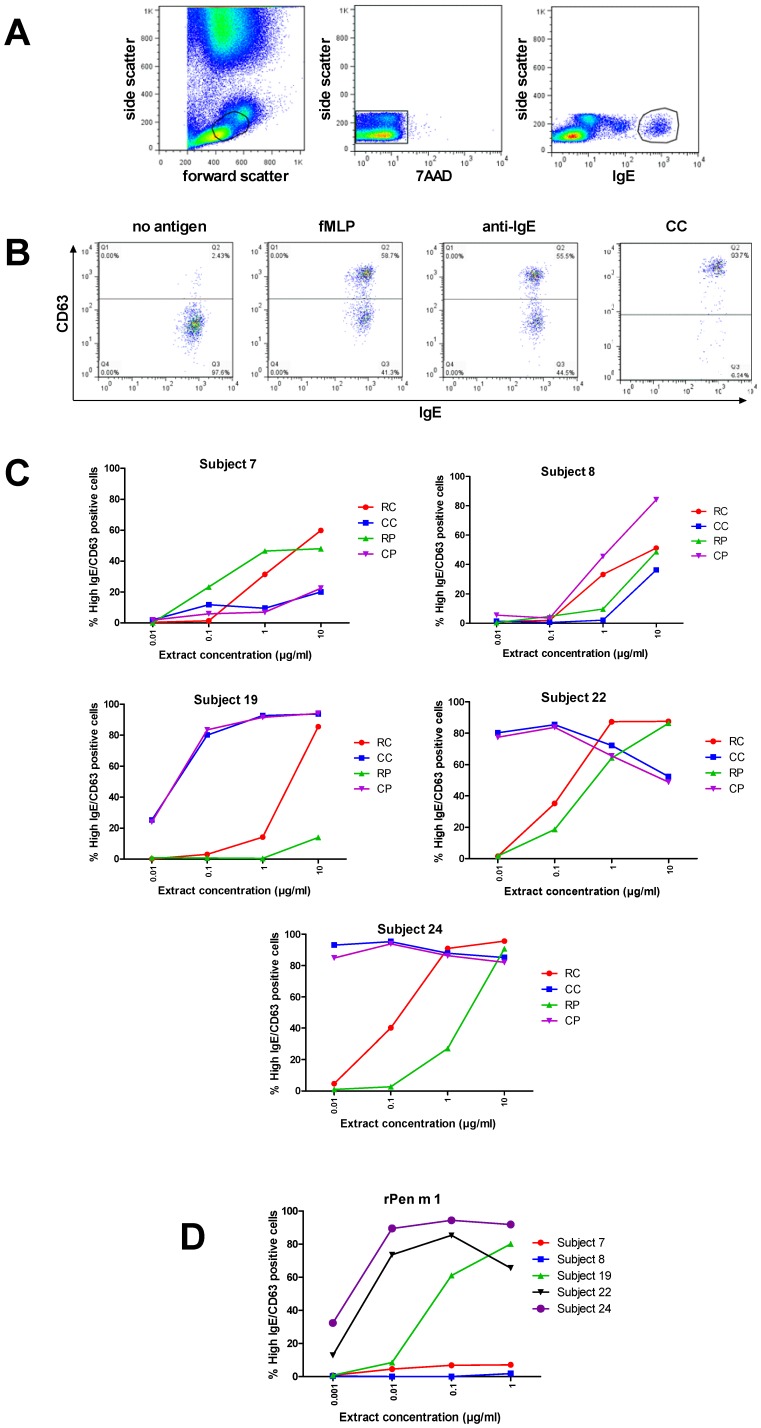
*In vitro* basophil activation by shellfish extracts and rPen m 1. **A.** Representative dot plots showing gating of viable basophils (7AAD^−^, high IgE positive). **B.** Representative dot plots (subject 19) showing analysis of activated basophils (up-regulation of cell surface CD63) for negative and positive controls, and CC (10 µg/mL). **C.** Dose-dependent activation of basophils from shellfish-allergic subjects (7, 8, 19, 22, 24) by shellfish extracts. **D.** Dose-dependent activation of basophils from shellfish-allergic subjects (7, 8, 19, 22, 24) by rPen m 1.

To assess biologically relevant shellfish allergen-specific IgE antibody reactivity, the ability of the different extracts to activate basophils from five shellfish-allergic subjects (7, 8, 19, 22, 24) was analyzed by flow cytometry. Activated basophils were identified by high IgE expression and up-regulation of surface CD63 (Figure 6A, B). No non-allergen specific activation of basophils or toxicity was caused by the different shellfish extracts, as determined by incubating extracts with the basophils from a non-shellfish allergic, atopic subject and a non-atopic subject (data not shown).

Dose-dependent basophil activation to the crab and prawn extracts was observed, with a range of sensitivities for the subjects, consistent with their different crab- and prawn-specific IgE reactivities by ImmunoCAP and our ELISA and immunoblotting assays (Figure 6C). When subject basophil sensitivities were compared by examining the extract concentration required for 50% maximal stimulation, three subjects (19, 22, 24) showed markedly higher basophil activation by the cooked extracts than the raw extracts, with little difference between the two crustacean species. Subjects 7 and 8 showed lower basophil activation with similar sensitivity to the four extracts. rPen m 1 was shown to induce strong basophil activation in those subjects with high reactivity to the whole extracts (Figure 6D).

### Inhibition IgE ELISA

Inhibition ELISA was used to quantitate the degree of IgE cross-reactivity between the two shellfish species and the effects of cooking. No non-specific inhibition of IgE reactivity by the shellfish extracts, rPen m 1 or rPor p 1 was evident using serum IgE from a Bahia grass pollen-sensitized control subject and Bahia grass pollen extract (data not shown). Using shellfish-allergic subjects, both cooked extracts showed greater inhibition of IgE binding than the corresponding raw extracts (Table 3). For most subjects, the lowest concentration of cooked extract (0.16 µg/mL) resulted in >50% inhibition of IgE binding to both CC and CP. RC was a more efficient inhibitor of IgE binding to the cooked extracts than RP. Both rPen m 1 and rPor p 1 inhibited >50% serum IgE reactivity to CC and CP at the lowest concentration (0.048 µg/mL) for all except three subjects (11, 19, 24). In these cases, higher concentrations were required, but these were still well below the maximum tested except for serum 11. It was noted that this serum showed marked IgE reactivity to allergens other than TM by immunoblotting.

**Table 3:** Inhibitor concentration (µg/mL) required for 50% inhibition of IgE binding to cooked blue swimmer crab (CC) extract or cooked black tiger prawn (CP) extract.

	Coating antigen: CC						Coating antigen: CP					
	Inhibitor						Inhibitor					
Subject	RC	CC	RP	CP	rPen m 1	rPor p 1	RC	CC	RP	CP	rPen m 1	rPor p 1
5	12	<0.16	>100	<0.16	<0.05	<0.05	60	0.64	>100	<0.16	<0.05	<0.05
11	2.4	<0.16	16	<0.16	1.2	>30	18	<0.16	20	<0.16	1.2	0.3
17	1.3	<0.16	36	<0.16	<0.05	<0.05	1.6	<0.16	47	<0.16	<0.05	<0.05
19	0.8	<0.16	60	<0.16	<0.05	<0.05	2.4	<0.16	47	<0.16	<0.05	0.12
21	1.3	<0.16	47	<0.16	<0.05	<0.05	2.9	<0.16	47	<0.16	<0.05	<0.05
23	4	<0.16	87	<0.16	<0.05	<0.05	20	<0.16	90	<0.16	<0.05	<0.05
24	>100	14	84	20	0.14	<0.05	2.1	<0.16	20	<0.16	<0.05	<0.05

### Inhibition IgE Immunoblotting

**Figure 7:**
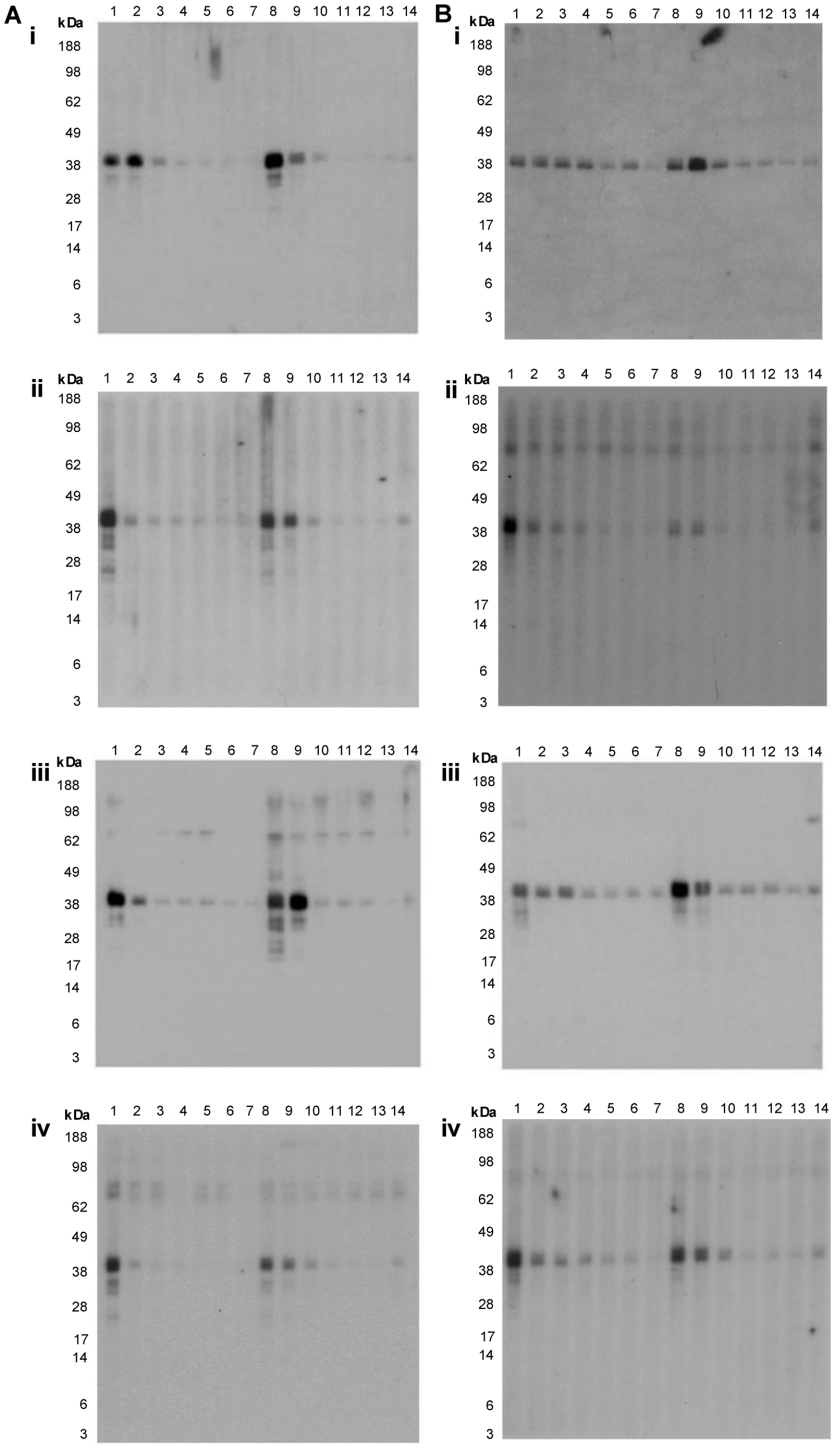
Inhibition IgE immunoblotting with shellfish extracts. Inhibition of serum IgE reactivity to CC (A) and CP (B) by shellfish extracts for subjects 5 (i), 19 (ii), 21 (iii) and 24 (iv). Lanes 1: No inhibitor, 2: 4 µg/ml RC, 3: 20 µg/ml RC, 4: 100 µg/ml RC, 5: 4 µg/ml CC, 6: 20 µg/ml CC, 7: 100 µg/ml CC, 8: 4 µg/ml RP, 9: 20 µg/ml RP, 10: 100 µg/ml RP, 11: 4 µg/ml CP, 12: 20 µg/ml CP, 13: 100 µg/ml CP, 14: 1.2 µg/ml rPen m 1.

Inhibition immunoblotting was used to examine the proteins responsible for cross-reactivity of IgE antibodies between the different extracts, and in particular whether there were potentially unique allergens within the blue swimmer crab. Again, non-specific inhibition by the extracts was excluded by testing for inhibition of the binding of serum IgE from a Bahia grass pollen-allergic control subject to Bahia grass pollen extract (data not shown). Four shellfish-allergic subjects (5, 19, 21 and 24) with IgE reactivity to TM (39 kDa) were selected for this assay. For all subjects, rPen m 1 markedly inhibited IgE binding to the TM band, further confirming the identity of this protein as TM (Figure 7). The CC and CP extracts also strongly inhibited IgE binding to TM in both species. However, the raw extracts inhibited IgE binding to TM only at higher concentrations, with RC showing greater inhibition than RP. Similarly, reactivity with the low MW proteins in both CC and CP was inhibited by the cooked extracts and RC, but poorly with RP. rPen m 1 could also inhibit the reactivity to the low MW bands, suggesting that they were predominantly breakdown products of TM.

**Figure 8:**
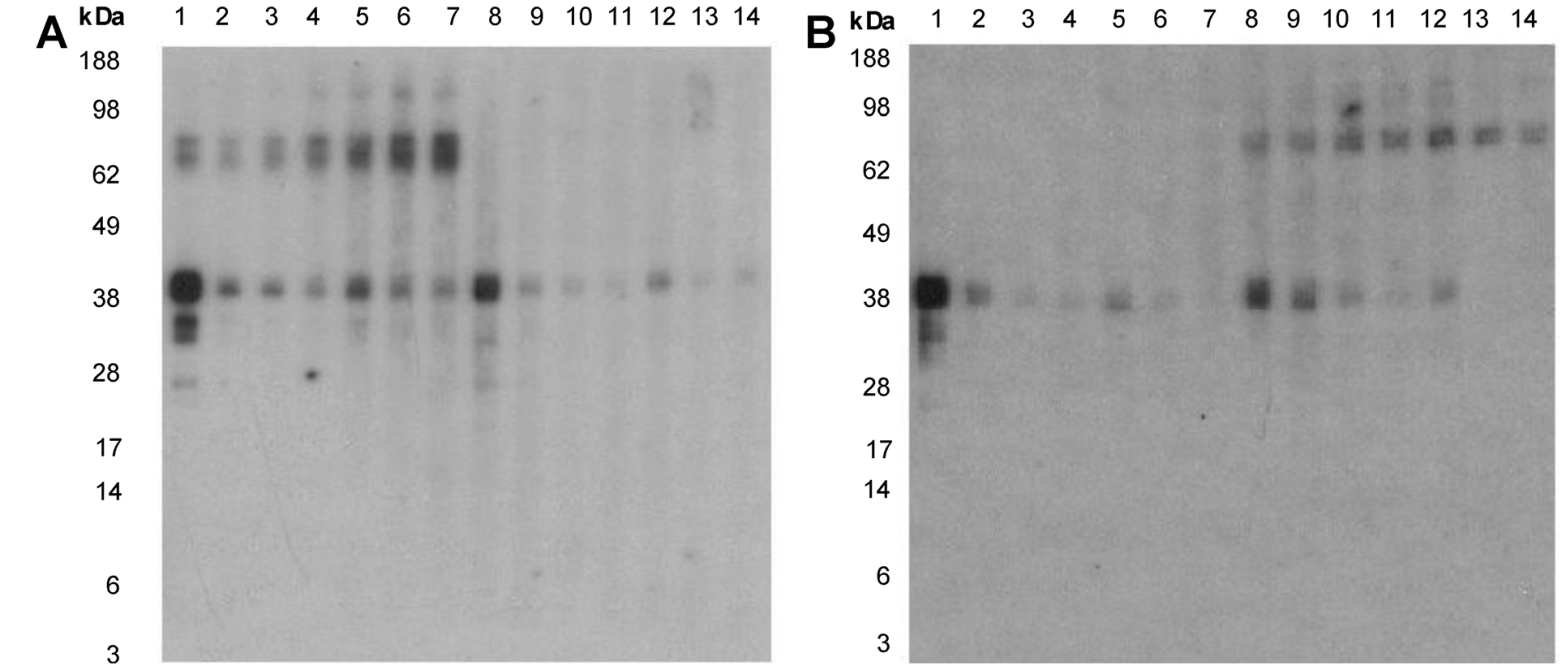
Inhibition IgE immunoblotting with recombinant tropomyosin preparations. Inhibition of serum IgE reactivity to CC (A) and CP (B) by rPor p 1 and rPen m 1 using subjects 24 (lanes 1–7) and 19 (lanes 8–14). Lanes 1: No inhibitor, subject 24, 2: 0.048 µg/ml rPor p 1, 3: 0.24 µg/ml rPor p 1, 4: 1.2 µg/ml rPor p 1, 5: 0.048 µg/ml rPen m 1, 6: 0.24 µg/ml rPen m 1, 7: 1.2 µg/ml rPen m 1, 8: No inhibitor, subject 19, 9: 0.048 µg/ml rPor p 1, 10: 0.24 µg/ml rPor p 1, 11: 1.2 µg/ml rPor p 1, 12: 0.048 µg/ml rPen m 1, 13: 0.24 µg/ml rPen m 1, 14: 1.2 µg/ml rPen m 1.

When the IgE inhibitory reactivity of rPor p 1 and rPen m 1 were compared over a concentration range using two shellfish-allergic patient sera, rPor p 1 showed slightly greater inhibition of IgE reactivity with the TM band in CC than rPen m 1, and rPen m 1 inhibited reactivity with CP TM slightly better than rPor p 1(Figure 8). Neither recombinant TM preparation inhibited IgE reactivity with the higher MW bands.

## Discussion

Shellfish allergy is a serious and increasing health issue, with current limitations in accurate diagnosis and management due to lack of information regarding clinically relevant allergens and IgE cross-reactivity between shellfish species. Crustaceans, especially crabs and prawns, are a common cause of shellfish allergy world-wide. This study examined the IgE-reactive components of a commonly eaten crustacean species, the blue swimmer crab, compared with those of a well characterized species, the black tiger prawn. In particular, the effects of heating on IgE reactivity and cross-reactivity of the crab allergens were investigated. In addition, the major TM allergen of the blue swimmer crab was cloned and sequenced and its IgE reactivity characterized.

When shellfish-allergic subject serum IgE reactivity with whole extracts was compared, the raw crab extract showed greater IgE reactivity compared with the raw prawn extract by both ELISA and immunoblotting. Whether this was due to inherent differences between the proteins of the blue swimmer crab and black tiger prawn, or to differences in sensitizing species or route of sensitization is not clear. We and others have shown previously that inhalation during commercial processing can result in sensitization to seafood [29]–[32]. In the present study, although some subjects identified food handling as a cause of adverse reaction, this route could not be distinguished in all subjects and the majority reported ingestion-related allergic episodes. More strikingly, we found that cooked extracts were more IgE reactive than raw for both species. This may be due to the more common ingestion of cooked crab and prawn or to chemical modification of the crustacean proteins on heating as discussed below. That the IgE reactivity of the crustacean extracts observed in our study was clinically relevant was demonstrated by the functional, basophil activation test, again confirming higher allergenicity of the cooked extracts.

IgE immunoblotting studies were performed to examine individual IgE-reactive proteins. As for other crustacean species, TM is a major allergen of the blue swimmer crab. Over 50% of shellfish-allergic subjects showed IgE-reactivity to proteins corresponding to TM in both raw and cooked crab extracts and TM identity was confirmed by TM-specific mAb reactivity and peptide mass fingerprinting of the highly IgE-reactive 39 kDa protein. We report here for the first time the cloning and full sequence analysis of the *Portunus pelagicus* TM, Por p 1. This revealed strong homology of the blue swimmer crab TM with other crustacean TMs, but regions of amino acid sequence difference at sites of known and predicted linear IgE epitopes support the need for crustacean species-specific diagnostic reagents. The purified rPor p 1 showed high IgE reactivity by direct immunoblotting, but in agreement with sequence differences, IgE inhibition immunoblotting showed differential reactivity of rPor p 1 and rPen m 1. When compared for inhibition of IgE reactivity with TM in shellfish extracts, rPor p 1 could better inhibit reactivity with the cooked blue swimmer crab TM and rPen m 1 was the better inhibitor of reactivity with black tiger prawn TM. Several other allergenic proteins of the blue swimmer crab were recognized at lower frequency. For some subjects, these proteins were recognized in the absence of TM reactivity. Testing of a larger subject cohort is required to assess the clinical importance and cross-reactivity of these proteins and hence define an appropriate panel of defined allergens for refined diagnostic assays.

In view of potential food matrix-associated effects on the outcome of heating of allergens [33], we chose to examine heating of whole extracts rather than purified allergens. For both the crab and prawn species studied, ELISA and immunoblotting showed markedly increased IgE reactivity of cooked extracts compared with raw. In particular, IgE immunoblotting demonstrated increased IgE-reactivity of TM within the cooked extracts. Although all sera including the non-atopic serum bound weakly to the TM band in the CP extract suggesting non-specific binding due to glycosylation for example, if this reactivity was used as a background reference, stronger binding of some shellfish-allergic sera was observed. The higher non-specific reactivity of the CP extract in this assay is consistent with the higher cut-off value for the IgE ELISA for this extract. Several higher MW bands with smearing, also typically a feature of glycosylated proteins, were observed in the cooked extracts. Some of these may represent heat-modified TM multimers. In addition, a range of highly IgE-reactive lower MW proteins was observed following heating, presumably largely TM fragments since this was especially notable for sera that reacted with the single TM band in the raw extracts. Investigation of the mechanisms responsible for the increase in allergenicity of TM and other allergens within the whole shellfish extracts following cooking is warranted as most shellfish is heat processed before consumption. Potential mechanisms include denaturation of protein with exposure of neo-epitopes and the Maillard reaction. In this heat-dependent reaction, sugars, both endogenous and exogenous, are non-enzymatically attached at different locations on the protein molecule generating advanced glycation end products [23], [34]. The Maillard reaction has not been well explored in the context of shellfish allergy, although found to play a role in the IgE reactivity of other allergens, particularly peanut allergens [35]–[36]. Our findings support the inclusion of thermally-processed whole extract as well as defined allergen preparations in commercial diagnostic tests for shellfish allergy.

Cross-reactivity between crustacean species is essential to understand in order for shellfish-allergic subjects to receive the best clinical advice on food avoidance. There are limited studies of crustacean cross-reactivity to date, often with small subject cohorts [15], [37]–[39]. Our study showed that IgE cross-reactivity between the blue swimmer crab and black tiger prawn was high, especially between the cooked extracts. Cross-reactivity between the cooked extracts was symmetric as both were able to effectively inhibit IgE binding to each other to a similar extent [40]. This means that the sensitizing species is unable to be determined in most cases without accurate clinical history. As shown by inhibition ELISA, except for serum 11, the capacity of cooked whole extracts to inhibit IgE binding to the cooked crab extract was similar to that for rPor p 1, consistent with allergenicity in the cooked blue swimmer crab being largely due to cross-reactive TM. TM has previously been documented as the major allergen of the black tiger prawn [9]. Our sequence analysis of the blue swimmer crab TM, Por p 1, and alignment with black tiger prawn TM, Pen m 1, provides a molecular basis for the high IgE cross-reactivity observed between these species in our study. The different reactivity seen with serum 11 is consistent with its stronger reactivity with a higher MW band than with TM by IgE immunoblotting.

Screening of shellfish-allergic subjects by IgE ELISA against raw and cooked extracts gave insight into whether the current diagnostic ImmunoCAP for crab is relevant in a southern hemisphere clinical setting. Although a double-blind placebo-controlled food challenge can confirm diagnosis of food allergy, this procedure has a high risk of anaphylaxis for adults with shellfish allergy who may have other comorbidities, and is not routinely performed in our hospital [41]. Similarly, skin prick testing of these patients is also not routine practice. For these reasons, in this study only ImmunoCAP data together with a careful clinical history were collected. In most cases, subjects were unable to identify clearly which individual crustacean species had provoked their clinical reaction. However, for the three subjects who did identify crab as an offending species (Table 1), only two of these had positive ImmunoCAP scores for crab-specific IgE. The third subject (14) had a negative crab ImmunoCAP but tested positive for both raw and cooked blue swimmer crab in our IgE ELISA. We found a significant correlation between the cooked, but not raw, crustacean extract ELISA results and ImmunoCAP scores, but in addition to the subject mentioned above, a small number of subjects with a negative crab ImmunoCAP result showed IgE reactivity to the crab extracts in both the IgE ELISA and immunoblot. This finding suggests either greater sensitivity of our assays, and/or lack of appropriate crab species or preparation method in the ImmunoCAP allergen preparation (*Cancer pagurus* or brown crab, a northern hemisphere species). All subjects with IgE-reactivity to crab or prawn TM by immunoblot had moderate to high levels of allergen-specific IgE (≥2.37 kU_A_/L) as determined by ImmunoCAP to shrimp and/or crab. These subjects were also more likely to have had severe allergic symptoms, such as angioedema and anaphylaxis, upon contact with shellfish. However, there were some subjects who had a strong ImmunoCAP result to shrimp and/or crab and clinical history of severe reactions but showed low or no IgE reactivity by ELISA or immunoblot. These subjects may have species-specific IgE with limited or no cross-reactivity with the crustacean species in our study [42]–[43], likely due to ingestion of different species.

Previous studies have concluded that specific-IgE to TM is an accurate predictor of shrimp allergy [44]–[45]. Our study supports the importance of TM as a major allergen of the blue swimmer crab, but several other crab proteins were shown to be allergenic and subjects exhibited different allergen reactivity profiles, several with no TM IgE reactivity. Component-resolved allergen-microarray technology would allow the simultaneous screening of serum IgE reactivity to a full panel of shellfish allergens, including whole allergen extracts, purified native and recombinant allergens, allergen fragments, and cooked and raw preparations. This would be of great advantage for the sensitive and specific diagnosis of shellfish allergy, and more information regarding the correlation between allergen-sensitization and severity of clinical symptoms could be gathered.

In conclusion, heating causes a marked increase in clinically relevant IgE reactivity of blue swimmer crab extract. In particular, we identified and characterized the blue swimmer crab TM, Por p 1, as a heat-stable major cross-reactive allergen. Other IgE-reactive blue swimmer crab proteins were observed, some corresponding to molecular weights of documented shellfish allergens, but others currently unidentified, some potentially unique to the blue swimmer crab. Our findings will advance reliable diagnosis and management of shellfish allergy.
